# Clinic Versus Online Social Network–Delivered Lifestyle Interventions: Protocol for the Get Social Noninferiority Randomized Controlled Trial

**DOI:** 10.2196/resprot.8068

**Published:** 2017-12-11

**Authors:** Monica L Wang, Molly E Waring, Danielle E Jake-Schoffman, Jessica L Oleski, Zachary Michaels, Jared M Goetz, Stephenie C Lemon, Yunsheng Ma, Sherry L Pagoto

**Affiliations:** ^1^ Department of Community Health Sciences Boston University School of Public Health Boston, MA United States; ^2^ Department of Allied Health Sciences College of Agriculture, Health, and Natural Resources University of Connecticut Storrs, CT United States; ^3^ Division of Preventive and Behavioral Medicine Department of Medicine University of Massachusetts Medical School Worcester, MA United States

**Keywords:** methods, randomized controlled trial, life style, obesity, weight loss, social support

## Abstract

**Background:**

Online social networks may be a promising modality to deliver lifestyle interventions by reducing cost and burden. Although online social networks have been integrated as one component of multimodality lifestyle interventions, no randomized trials to date have compared a lifestyle intervention delivered entirely via online social network with a traditional clinic-delivered intervention.

**Objective:**

This paper describes the design and methods of a noninferiority randomized controlled trial, testing (1) whether a lifestyle intervention delivered entirely through an online social network would produce weight loss that would not be appreciably worse than that induced by a traditional clinic-based lifestyle intervention among overweight and obese adults and (2) whether the former would do so at a lower cost.

**Methods:**

Adults with body mass index (BMI) between 27 and 45 kg/m^2^ (N=328) will be recruited from the communities in central Massachusetts. These overweight or obese adults will be randomized to two conditions: a lifestyle intervention delivered entirely via the online social network Twitter (Get Social condition) and an in-person group-based lifestyle intervention (Traditional condition) among overweight and obese adults. Measures will be obtained at baseline, 6 months, and 12 months after randomization. The primary noninferiority outcome is percentage weight loss at 12 months. Secondary noninferiority outcomes include dietary intake and moderate intensity physical activity at 12 months. Our secondary aim is to compare the conditions on cost. Exploratory outcomes include treatment retention, acceptability, and burden. Finally, we will explore predictors of weight loss in the online social network condition.

**Results:**

The final wave of data collection is expected to conclude in June 2019. Data analysis will take place in the months following and is expected to be complete in September 2019.

**Conclusions:**

Findings will extend the literature by revealing whether delivering a lifestyle intervention via an online social network is an effective alternative to the traditional modality of clinic visits, given the former might be more scalable and feasible to implement in settings that cannot support clinic-based models.

**Trial Registration:**

ClinicalTrials.gov NCT02646618; https://clinicaltrials.gov/ct2/show/NCT02646618 (Archived by WebCite at http://www.webcitation.org/6v20waTFW)

## Introduction

Lifestyle interventions have had established efficacy for over a decade but are not widely disseminated largely due to high cost and burden to patients and providers [1]. Online social networks provide an alternative way to interact with, educate, and counsel patients and may be less expensive because clinic visits are the main source of cost and burden in traditional lifestyle interventions [2-5]. Another advantage of online social networks is their capacity to reach large segments of the population without geographical barriers [6]. The majority of US adults who access the Web (79%) have at least one social network account and 56% have accounts on multiple platforms [7]. Data from the Pew Research Center indicates that Twitter is one of the most ethnically diverse online social network platforms; among adult Internet users, 21% of whites, 25% of Latinos, and 27% of blacks report using Twitter [8]. Additionally, many online social network users use these platforms to seek health information and connect with users with similar health concerns [9]. Thus, online social networks represent a promising modality to deliver lifestyle interventions and reach larger populations as well as vulnerable and underserved subgroups.

A growing number of studies have explored using online social networks, such as Facebook [10-12], Twitter [2,13,14], or study-specific online social networks [15,16], as a primary modality to deliver lifestyle interventions. A systematic review of 5 completed studies found weight loss ranging from −0.63 to −5.0 kg over 8 to 24 weeks [4]. A randomized feasibility trial of obese adults (N=70) found that a 6-month Facebook-delivered weight loss intervention was associated with similar weight loss compared with a group conference call-delivered intervention (5.8 kg vs 6.3 kg over 6 months) [10]; however, nonsignificance cannot be assumed to mean noninferiority because the study was not powered to test noninferiority. A series of pilot studies using private Twitter groups to deliver a lifestyle intervention found that this approach was feasible and acceptable, with a mean weight loss of 2.5 kg (3.0%) in 12 weeks [2]. If an online social network–delivered lifestyle intervention resulted in weight loss that was not appreciably worse than the traditional clinic-delivered model, it would provide an option for settings in which clinic-based interventions are not feasible.

The purpose of this paper is to describe the design and methods of a noninferiority randomized controlled trial (RCT) comparing a lifestyle intervention delivered entirely via an online social network with a traditional in-person clinic-based lifestyle intervention among overweight and obese adults. Intervention strategies in both study conditions are based on the Diabetes Prevention Program (DPP), which is considered the gold standard in evidence-based behavioral weight loss programs [17-19] and has been replicated and adapted numerous times [20]. Furthermore, the translational potential and effectiveness of the DPP have been evaluated across several studies. A systematic review and meta-analysis of 28 US-based studies that tested DPP-based interventions in real-world settings indicated an average of 4% weight loss at 12 months [20]. Intervention attendance varied, with the number of core DPP sessions offered positively correlating with the number of core sessions attended, and low attrition rates (10%-16%) were observed among studies that implemented the DPP using online or digital versatile disc (DVD) formats [20]. We hypothesize that the social network-delivered intervention will not be appreciably worse than the traditional clinic-delivered intervention in weight loss at 12 months. Secondary noninferiority outcomes are changes in dietary intake and physical activity from baseline to 12 months. We also hypothesize that the social network–delivered intervention will cost less per participant to implement than the clinic-based intervention. Exploratory outcomes include treatment retention, acceptability, and burden. We hypothesize that the online social network condition will have better retention and acceptability, with lower participant and clinician burden. We will also explore predictors of weight loss in the online social network condition. We hypothesize that greater engagement, younger age, higher sociability, neuroticism, openness, and greater social network use will be associated with greater weight loss in the online social network condition.

## Methods

### Study Design

This study is a noninferiority RCT comparing a lifestyle intervention delivered entirely via the online social network Twitter (Get Social condition) with an in-person group-based lifestyle intervention (Traditional condition) among overweight and obese adults. The use of the noninferiority design provides enough statistical power to establish that one modality is not appreciably worse than another [21].

### Study Population

A total of 328 overweight or obese adults will be randomized to the two conditions. To be eligible for participation, participants must meet the following inclusion criteria: (1) a body mass index (BMI) between 27.0 and 45.0 kg/m^2^, (2) aged between 18 and 65 years, (3) written clearance from their primary care providers, (4) possession of a smartphone, (5) an active social media user (ie, currently have an online social network account and log in at least 4 days/week), and (6) an interest in losing weight. Participants with a personal Twitter account will be asked to set up a separate Twitter account to use for participating in the study. Twitter allows users to have multiple accounts, and linked accounts can be toggled back and forth with a click on the Twitter app.

Individuals who meet any of the following criteria will be excluded from study participation: (1) plans to move during study, (2) pregnant, lactating, or plans to become pregnant during the study, (3) taking medication that influences weight, (4) participating in a formal weight loss program (eg, Weight Watchers), (5) lost 5% or more weight in the past 3 months, (6) bariatric surgery or plans to undergo surgery during the study period, (7) a medical condition that precludes dietary or physical activity changes, (8) type 1 diabetes reported by the participant or their primary care provider or uncontrolled type 2 diabetes determined by the participant’s primary care provider in a medical clearance letter, (9) currently smoking more than 3 cigarettes per day, and (10) preference for one condition over the other. Participants who do not complete baseline measures, do not agree to be randomized, and do not participate in a study orientation webinar will also be excluded.

### Participant Recruitment

Study participants will be recruited from the local Central Massachusetts area in 9 waves of 36 to 37 participants. Participants will be recruited from the community, including online recruitment sources such as Craigslist, Twitter, postings in local Facebook groups (eg, local parent groups), the University of Massachusetts Medical School and UMass Memorial Health Care employee and student intranet, online newsletters, print newspapers, mass emails, and flyers in local community locations. We will also recruit participants through paid ads on Google, Facebook, and Twitter. Some recruitment efforts will specifically target males and racial/ethnic minorities, given that previous weight loss intervention trials underrepresent these groups [22]. Targeted recruitment efforts include recruiting participants from Facebook groups that have a high rate of male or racial/ethnic minority presence, recruiting participants from local businesses that have a high racial/ethnic minority population or that serve male clientele, and engaging a community leader to serve as a consultant on the study to aid in racial/ethnic minority recruitment efforts. Interested participants will email or call study staff to complete a telephone screening call, which will consist of a review of information about the study and an eligibility assessment. Eligible participants will be scheduled for a 1.5-hour baseline visit, which will include a review of written informed consent, physical measurements, a baseline survey, and the first of three computer-based 24-hour dietary recalls. Participants will be asked to provide the name of their primary care provider so he/she can be contacted for medical clearance. Participants will be asked to complete a randomization agreement that first explains randomization and asks participants specific questions about their understanding of each condition. Finally, participants will complete two additional computer-based dietary recalls at home over the following week. Participants will be provided US $30 after completing the baseline assessment.

### Study Orientation Webinar

Before randomization and after completing the baseline assessment, participants will attend a 1-hour orientation webinar, adapted from the model used successfully in two other previous completed weight loss trials [23,24]. During the webinar, a study staff member will describe all aspects of the study, what it means to participate in research, what it means to be randomized, the importance of follow-up data to the integrity of the study, and what study participation entails. Attendees will have the opportunity to ask questions and to discuss the pros and cons of participation.

### Randomization

Each wave of participants will be randomized 1:1 to the two study conditions in randomly permuted blocks of sizes 4 and 6 using the ralloc program in Stata (Stata Corp) [25]. Randomization will be stratified by gender (male vs female) and baseline BMI (27.0-34.9 kg/m^2^ vs 35.0-45.0 kg/m^2^).

### Orientation Visits

#### Get Social Condition

Twitter was selected as the online social media platform for intervention delivery for several reasons. First, using a commercial platform saves the cost of developing a separate platform. Second, only 24% of the population uses Twitter, so most participants will not be users, unlike Facebook on which 79% of the population has a Facebook account [7]. To the extent that users utilize one of their personal social media accounts for the study, the intervention material will compete with the rest of the users’ newsfeed, which could affect treatment receipt even among highly motivated users. In terms of having a separate account for the study, Facebook discourages single users having multiple accounts and has features that make it difficult to do so (eg, cannot use the same name or phone number on two accounts). Twitter makes it easy for users to have two accounts, and once accounts are linked, users can just click a button on the Twitter app to move from account to account without logging out of one and into the other. Finally, Twitter has very simple privacy settings (either all private or all public) which helps quell privacy concerns that arise in the context of complex, often changing privacy settings.

Participants randomized to the Get Social condition will attend individual and group orientation visits before the intervention. Given that all participants are locally recruited and could be randomized to either the Get Social or Traditional condition, we opted for in-person orientation visits to ensure that participants understand how to use the technology, though conducting the orientation visit via webinar may be a preferred method for future effectiveness trials.

At the individual orientation visit, participants will learn how to set up and use a private Twitter account. Twitter’s privacy setting protects one’s tweets from being viewed by anyone except those approved by the user. All participants and the dietitian use the “protected” privacy setting so that all interactions are only viewable to each other, thus creating a private group on Twitter. Participants are informed that their followers can see their username, Twitter handle, and profile picture. They will be urged to select aliases and avatars to represent themselves in the biographical section of their account to protect their anonymity. At start-up, participants will be asked to hold off on following nonstudy accounts so that their newsfeed is exclusively for the group. However, as the program posts become less frequent in the later phases of the intervention, they will be given suggestions for evidence-based healthy lifestyle feeds to follow to grow their social network. Participants will be advised not to post anything they feel uncomfortable posting and to remain on topic. Participants who wish to have a private exchange with a dietitian or a fellow participant will be instructed to use the Twitter direct message feature, which allows private one-on-one communication between users. Participants will also be oriented to the study blog and instructed to log in with all devices they use regularly so that their time spent on the blog can be tracked for the cost analyses. The study blog will contain papers that dietitian posts occasionally, with a link to provide more information on a topic.

At the group orientation, participants will follow the dietitian and all other participants in their group on Twitter will receive instruction on ways to participate and practice tweeting. They will receive instruction on how to get the most out of the intervention, including guidelines to read the daily posts by the dietitian, to read and reply to each other’s posts, to post about their progress and challenges, and to ask the dietitian or group any questions that come to mind. They will also learn about the DPP lifestyle intervention, program goals for weight loss and physical activity, and how to use MyFitnessPal for dietary and exercise self-monitoring. A mobile app for tracking is necessary in the Get Social condition because dietitians will not have physical contact with participants to exchange paper records. Instead, they can access participants’ data by logging into their account. Protocols for the individual and orientation visits were developed based on a series of pilot studies [2].

#### Traditional Condition

Participants in the Traditional condition will attend a group orientation visit. At this visit, participants will learn about the DPP lifestyle intervention [26] program goals for weight loss and physical activity, as well as how to use MyFitnessPal for dietary and exercise self-monitoring. To avoid an imbalance across conditions in terms of mobile app versus paper-and-pencil tracking, participants in the Traditional condition will also be encouraged to use MyFitnessPal to track their diet and activity. However, participants preferring paper-and-pencil tracking or use of a different app will be allowed to use these instead.

### Study Conditions

Participants in both study conditions will receive a 12-month lifestyle intervention based on the DPP. The DPP includes instruction in self-monitoring of food intake, nutrition, exercise, and behavioral modification [26]. The DPP was chosen for this study, given the ample efficacy data for weight loss and disease prevention [27-31]. Three dietitians who received training in both conditions will be randomly assigned to one condition each wave, alternating between conditions. By the end of the study, each dietitian will have led the same number of groups for the online social network and in-person modalities.

#### Get Social Condition

Participants randomized to the Get Social condition will receive 12 months of lifestyle counseling via a private Twitter group. For months 1 to 2 of the intervention, the dietitian account will post twice daily, for months 3 to 6 the dietitian account will post once daily, and for months 7 to 12, the dietitian account will post 4 times weekly. The frequency and spacing of these interactions correspond to the frequency and spacing of in-person visits that occur in the DPP.

Each DPP session was distilled into a collection of 14 tweets that met all visit objectives [32,33]. Tweets are of the following types: text only; a combination of text and an image of an excerpt of the original materials; a combination of text and an image that reflects the content of the text, polls, and text; and a link to a study blog post or other online resource that elaborates on the topic. The latter are used for more complex topics. The study blog was created with free blogging software, and blog posts were created in the format and length that is commonly seen in patient-oriented blogs (eg, 800 words with images). Content tweets will come from the dietitian’s feed. To reduce interventionist burden, the tweets are automated and prescheduled to appear at 5 AM and 4 PM twice daily on weekdays and 5 AM once daily on weekends. The dietitian then logs in twice daily to field responses to the automated posts, answers and poses questions, tweets “check ins” to participants who have not engaged recently, replies to participants, “likes” participants’ replies and posts, and replies to private direct messages.

Every Friday morning post will ask participants to reply to report their weight change from the previous week. This approximates the “weigh-ins” that occur in the Traditional condition, while protecting their privacy by focusing on change in weight from the previous week (eg, +1 lb and −1 lb) rather than absolute weight. To encourage participants to check their Twitter feed, participants will be informed of the various ways they can receive notifications (eg, emails and pop-ups) and will be encouraged to set notifications of their preference. They will be advised to log in daily to read the counselor’s posts and to engage with the group. Each week, a newsletter will be emailed to participants to encourage participants to engage with the group and to highlight some of the newsfeed from that week in case any participants miss the content when it is posted.

#### Traditional Condition

Participants randomized to the Traditional condition will receive 12 months of lifestyle counseling via clinic-based group meetings lasting 90 min per session. Participants will receive the Core of the DPP Lifestyle Intervention Core intervention for 6 months, followed by monthly group meetings for another 6 months (total of 22 sessions) [26]. For months 1 to 4, groups will meet weekly; for months 5 to 6, groups will meet biweekly; and for months 7 to 12, groups will meet monthly. Before each session, the dietitian will privately weigh each participant. Participants who miss groups will be emailed the session content.

### Follow-up Assessments

At 6 and 12 months, participants will complete an in-person study visit. Follow-up assessments include physical measurements, a follow-up survey, and the first of 3 computer-based 24-hour dietary recalls. Two additional computer-based dietary recalls will be completed randomly at home over the following week. Participants will receive US $40 after completing the 6-month follow-up assessment and US $60 after completing the 12-month assessment.

### Measures

#### Primary Noninferiority Outcome: Percentage Weight Loss

Trained personnel will measure participants’ height and weight using a digital scale and stadiometer with the participant wearing light clothing and no shoes; measurements will be taken to 2/10th of the nearest inch or pound. Percentage weight loss at 6 and 12 months will be calculated by subtracting follow-up weight from baseline weight divided by baseline weight.

#### Secondary Noninferiority Outcomes: Energy Intake and Physical Activity

To assess energy intake, participants will complete three 24-hour diet recall interviews over 2 weeks surrounding baseline, 6-, and 12-month in-person study visit using the National Cancer Institute’s (NCI) automated self-administered 24-hour dietary recall (ASA24) [34]. One recall will be done at the baseline assessment visit so that the participant can learn the program and be assisted if needed. The remaining two baseline recalls will be completed at home on randomly selected days (2 weekdays and 1 weekend day). The 6- and 12-month assessments will be completed in the same manner. Daily energy intake and other nutrients for each recall will be estimated using the United States Department of Agriculture’s Food and Nutrient Database, and the average of the three recalls will be used at each study visit.

To assess physical activity, participants will complete the 74-item Arizona Activity Frequency Questionnaire [35] at baseline, 6, and 12 months. Output will be calculated in number of minutes per day of moderate or higher intensity activity. Questionnaire assessments will be completed online using REDCap. The online link will be sent to participants to complete at home, and it will take approximately 1 hour to complete.

#### Cost

We will systematically track costs associated with delivery of both intervention conditions, capturing information on the costs that would be required to implement each intervention in practice (ie, outside the research context), including administrative, interventionists, and participant costs [36-38]. Time and other costs related to tasks performed to develop the interventions or to carry out the research, such as recruitment and study assessments, will not be included, as our interest is in comparing how much it would cost to deliver each intervention in real-world settings.

### Administrative Costs

We have created an online survey system that will be used to evaluate staff time and money spent on these activities. Staff will record how much time they spent on each task weekly and any monetary costs (eg, purchase of supplies and copying expenses). In the Get Social condition, administrative tasks include scheduling posts, software (Photoshop and Buffer) for modifying and posting tweets, orientation materials, and conducting the orientation visits. In the Traditional condition, administrative tasks include the purchase or printing of participant materials, sending materials to members who missed the group, orientation materials, and conducting the orientation visits. For staff and interventionist time, we will calculate costs based on actual staff salaries [39] and will conduct sensitivity analyses using national salary data.

### Interventionist Costs

Interventionists will report time weekly on a spreadsheet and document each task completed and the time taken to perform each task. Get Social condition interventionist tasks include time spent on the following: reading and responding to participant posts, fielding direct messages from participants, answering questions/concerns or researching information to answer questions, emailing participants who do not post for 2 weeks, and reviewing participant diet diaries. In the Traditional condition, interventionist tasks include time spent on the following: travel for group meetings, leading the group meetings, emailing participants who do not attend the group meetings, answering questions/concerns or researching information to answer questions, and reviewing participant diet diaries. Interventionists will record time spent on each task daily. Estimates for the cost of interventionist time spent traveling round trip to group meetings will be calculated as 5 min/mile for a 5-mile radius from the research center and then 2 min/mile beyond 5 miles based on local traffic patterns around campus.

### Participant Costs

Get Social condition participant tasks include the time spent on Twitter to participate in the intervention and time spent reading the study blog. On the weeks the Traditional condition has group meetings, we will contact participants in the Get Social condition via email requesting them to complete a self-report online survey. In these surveys, we will ask participants to report the time they spent on Twitter to participate in the intervention that week and then use these data to estimate total time across all weeks. Participants with iPhones will be asked to report the time spent on the Twitter app under battery usage settings. To assess the time a participant spent on the Twitter app specifically for intervention participation (rather than using the Twitter app for other reasons), we will ask participants whether they access the intervention feed using another device (such as a desktop or a laptop computer or a tablet), and what percentage of the time they spent using the Twitter app was to participate in the intervention, and adjust time estimates from the iPhone battery statistics on their responses. Participants with Android or Windows smartphones will be asked to self-report their time spent on Twitter. Time spent on the study blog will be tracked through Pardot, a company that provides analytics for customers’ online marketing campaigns. We will download these data from Pardot every 6 months.

Although participants will register their devices on the blog at baseline, it is possible that participants will access the blog from nonregistered devices. Therefore, the weekly surveys will also ask participants to report how many of the articles that the coach tweeted in the past 7 days they read, and we will compare these self-reported data with data downloaded from Pardot. In the Traditional condition, participant tasks include travel to and from the group meetings from their home address (using the same approach as for interventionist travel costs) and time spent attending group meetings. Time at group meetings for each participant will be based on the duration of each meeting recorded by the interventionist and attendance records. Participant time will be converted to costs using information about participant income [40]. In the absence of income data, we will use the median or average wages for adults in their state of residence (Massachusetts) [41]. We will conduct sensitivity analyses using national salary data. This approach allows us to estimate the cost of participating in the intervention for adults across the United States.

#### Exploratory Outcomes

##### Treatment Retention

We will track which participants drop out of treatment. We have a protocol for reengaging participants who are not actively participating in treatment. In the Get Social condition, staff tweet participants who have not engaged that week and ask them how they are doing. Following the second consecutive week with no visible online engagement, the coach will email the participant. If another week passes with no engagement on Twitter, the coach will call the participant. In the Traditional condition, staff will email the missed intervention materials to participants who miss the group session. Following the second consecutive missed group session, the coach will email the participant. If another group session is missed, the coach will call the participant. We will consider participants who have not engaged in treatment in 4 consecutive weeks and have not responded to our attempts to reengage, as well as participants who express wanting to withdraw from the intervention to have dropped out of treatment.

##### Acceptability and Burden

At 6 and 12 months, participants in both study conditions will rate the *acceptability* (easy, would be willing to do again, willing to continue, and comfort level) and *burden* (time-consuming and costly) of their treatment condition on 5-point Likert scales.

#### Predictors of Weight Loss in the Get Social Condition

We will explore predictors of weight loss in the online social network condition, including engagement, age, sociability, neuroticism, openness, and social network use.

##### Engagement

Engagement data on tweets by interventionists and participants will be downloaded weekly from Twitonomy, a Twitter analytics and monitoring tool. Information on “likes” for each tweet will be downloaded weekly via a script that captures the data from Twitter. We will calculate the number of original tweets, replies, and likes per participant. We will also sum these metrics for a total summary measure of engagement. At 6 and 12 months, we will also ask participants survey questions about lurking (reading without visibly interacting) [42] and calculate metrics of engagement that include lurking.

##### Age

Participants will report their age at eligibility screening.

##### Sociability, Neuroticism, and Openness

Participants will complete the Ten-Item Personality Inventory (TIPI) [43] at baseline. Sociability, neuroticism, and openness scale scores will be calculated for each participant.

##### Social Network Use

At baseline, participants report which online social networks they have accounts on and frequency of online social network use in the past 4 weeks (study survey). Online social network use is expected to be a predictor of weight loss in the Get Social condition because individuals who use online social networks more regularly may be more likely to lose weight than nonregular users as they are more accustomed to using these platforms and engaging online.

##### Contamination, Treatment Fidelity, and Participant Safety

###### Contamination

Contamination is defined as the use of other forms of online or in-person weight loss support in either condition during the study. Using the Pew Internet & American Life Project Poll [44] questions, we will evaluate and report the number of participants who received support for weight loss using online or in-person programs, blogs, pages, or connecting with participants.

###### Treatment Fidelity

We randomly selected one topic within each phase of the study (weeks 1-8, weeks 9-24, and weeks 25-52) for a total of 3 topics (10% of 22 topics). Each topic has the same objectives to meet in both conditions. In the Traditional condition, we have an independent reviewer listen to the recording of the selected group to ensure that each objective was met. In the Get Social condition, the topics span from 1 week to 4 weeks depending on the phase of the study (1 week in phase 1, 2 weeks in phase 2, and 4 weeks in phase 3). The respective weeks of the topic selected will be extracted from Twitter and reviewed by an independent reviewer to ensure each objective was met. Findings will be reported.

###### Participant Safety

Possible risks during the intervention include injury during exercise or breach of confidentiality. Participants who report conditions at baseline that could create a safety concern while receiving the intervention are excluded. To avoid possible risks, participants are instructed to avoid over exercising at intense levels that could lead to discomfort, pain, or injury. Participants reporting discomfort will be referred to their primary care providers. Participant data are stored in network secure data entry programs, and any data on paper are stored in a locked file cabinet. Adverse events that occur during the intervention are assessed, recorded, and followed up until resolved. Serious adverse events are communicated immediately to the data safety monitoring board and the institutional review board (IRB).

### Power Calculation

We powered the study to be able to detect noninferiority [21] for the primary outcome and percentage weight loss at 12 months. In a noninferiority trial, the null hypothesis (H_0_) is that the new treatment is inferior to the standard treatment, and the alternative hypothesis (H_A_) is that the new treatment is not inferior to the standard treatment. “Not inferior to” is defined by the noninferiority margin, δ. Other noninferiority behavioral weight loss trials have utilized various noninferiority limits depending on the study population, primary outcome, and timeline. One study examined BMI reduction among adolescents and set δ=0.12 based on a 0.21 reduction of BMI at 12 months [45], whereas another weight loss trial among adults set δ=at 1 kg at 3 months [46]. We set δ=2% as a relative margin to the 5% weight loss as clinically meaningful cut point [47] and because 2% is not so small a difference in average weight loss between study conditions that we would have to recruit a prohibitively large sample. This means that if the Get Social condition loses up to 2% less weight on average than the Traditional condition, we will consider the Get Social condition to be noninferior to the Traditional condition. Thus, adequate power for clinical noninferiority requires a sample size such that there is better than 90% probability that the lower limit of the CI lies above –δ, if the true effect size is zero or above. We estimated standard deviation (SD) of 5.5% based on a previous, fully powered randomized trial [48]. With alpha=.05 and δ=2%, we have 90% power to conclude that the Get Social condition is not inferior to the Traditional condition with 131 participants per arm. Accounting for 20% attrition, we will enroll 328 participants in total (164 per arm). We considered adjusting the sample for intraclass correlation, but previous research shows that weight loss does not cluster among members of weight loss treatment groups [49].

For secondary noninferiority outcomes, we used 90% power to calculate noninferiority margins given N=131 per arm, setting alpha=.05 and using observed SDs from the literature. For change in energy (kcal/day) intake at 12 months, the study is powered at 90% to detect that the Get Social condition is not inferior to the Traditional condition with a noninferiority margin of 182 kcal/day (SD=500 kcal/day) [48,50]. For change in moderate to vigorous intensity physical activity minutes per day at 12 months, the noninferiority margin is 9.2 min/day (SD=25 min/day). For percentage weight loss at 12 months, the noninferiority margin is 2.2% (SD=5.5%) [48].

With N=131 available per arm (N=262 total) and alpha=.05, we have 80% power to detect differences in mean cost per participant of 0.35 SDs. For example, if the SD for cost is US $100, then we have 80% power to detect differences in mean cost per participant of US $35. With N=131 available in the Get Social condition and alpha=0.05, we have 80% power to detect correlations of .243 between continuous predictors and 12-month percentage weight loss. For categorical variables, detectable differences in mean percentage weight loss depend on the proportion of the predictor in the sample. For a predictor with 50% prevalence (ie, n=65 and n=66 with vs without the characteristic), we have 80% power to detect differences of 0.49 SDs. For predictors with 33% prevalence, we can detect differences of 0.52 SDs. For predictors with 20% prevalence, we can detect differences of 0.62 SDs, and for predictors with 10% prevalence (ie, n=13 vs n=118), we can detect differences of 0.82 SDs. For estimated SD of 5.5% for 12-month percentage weight loss, this indicates differences of 2.7%, 2.9%, 3.4%, and 4.5% weight loss.

Power calculations for noninferiority analyses were conducted using PROC POWER in SAS 9.3 (SAS Institute, Cary, NC) [51,52] and power calculations for Aims 2 and 3 were conducted in Stata 13 (Stata Corp, College Station, TX).

### Analysis Plan

Reporting and data analyses of this trial will follow the recommendation of the 2012 *JAMA* paper *Reporting of Noninferiority and Equivalence Randomized Trials Extension of the CONSORT 2010 Statement* [53]. Analyses will be intent-to-treat, meaning all randomized participants will be analyzed and in their originally randomized conditions.

#### Preliminary Analysis

Baseline participant characteristics will be examined by condition. If groups differ on any characteristics, these variables will be included as covariates in the primary analyses. Other preliminary analyses will include assessing patterns of missing data, dropout rates, distributional properties of dependent measures, and correlations among outcome measures. A series of sensitivity analyses will be performed to examine the extent of potential bias by assuming that the participants who dropped out are missing completely at random (ie, independent of the outcome), are responders to the intervention, or are nonresponders to the intervention. Multiple imputations to impute missing data will be used if more than 5% of data are missing [54].

#### Primary Noninferiority Outcome

We will model percentage weight loss at 12 months using a linear regression model framework, with percentage weight loss as the dependent variable and study condition as the independent variable. Test of the intervention condition indicator will provide a statistical test of the intervention effect and the estimated coefficient, along with the estimated confidence interval. This analytic approach aims to test whether the Get Social condition is not appreciably worse than (ie, not inferior to) the Traditional condition by our a priori inferiority margin of 2%. The effect size estimates will reveal clinical noninferiority of the Get Social condition if the CI lies completely above −Δ or clinical noninferiority of the Traditional condition if the CI lies completely below +Δ, that is, 2%.

#### Secondary Noninferiority Outcomes

Secondary noninferiority outcomes (dietary intake and physical activity at 12 months) will be examined using the same approach as described for the primary outcome. Linear multivariable regression models will be used to estimate change in daily caloric intake at 12 months; such models are reasonable, given the target sample size and that changes in these outcomes are approximately normally distributed [48,55]. We anticipate using log transformation to estimate change in physical activity variables, given that physical activity data tend to be skewed. The distribution of secondary outcomes will be explored graphically and inform the primary outcome analysis; analyses will be modified as needed through transformation of the data.

#### Cost

We will compare total intervention costs per participant and total intervention costs per pound lost by treatment condition. As Ritzwoller and colleagues recommend [37,40], we will conduct sensitivity analyses to estimate the range of costs after varying the inputs (eg, time spent on Twitter by participants, staff pay across settings, and based on qualifications and training). We will also examine administrative, interventionist, and participant costs by treatment condition. Assuming a normal distribution of total costs per participant, we will first compute *t* tests comparing the average cost per participant across treatment conditions. We will test the null hypothesis of no difference between groups using a two-sided test and alpha=.05. If total cost per participant is not normally distributed, a nonparametric approach using the Mann-Whitney test for median comparisons will be used. If participant characteristics are found to differ according to treatment allocation/condition, multivariable linear regression models will be used to adjust for the potential confounding effects of these characteristics. Assuming a normal distribution of total costs per participant, multivariable linear regression models will be used. If not normal, we will identify appropriate transformations (eg, logarithm and square root) for the cost variable.

#### Exploratory Outcomes (Treatment Retention, Acceptability, and Burden)

We will compare treatment retention (percentage retained in treatment, ie, percentage who has not dropped out of treatment) and ratings of acceptability and burden (measured on 5-point Likert scales) across treatment conditions using chi-square tests.

#### Predictors of Weight Loss in the Get Social Condition (Exploratory)

Bivariate associations between each potential predictor of weight loss (eg, engagement, age, sociability, neuroticism, openness, and online social network use) and percentage weight loss at 12 months will be examined among participants in the Get Social condition using linear regression models. Multivariable predictors of percentage weight loss at 12 months will be estimated using linear regression models. Variables will be added to the model one at a time, in order of magnitude of the crude effect estimate (largest to smallest; *P*<.05) associated with the outcome. The final model will be selected based on consideration of effect estimates, 95% CIs, and Akaike information criterion.

## Results

The first wave of the intervention began in August 2016. Recruitment will continue through May 2018, and we anticipate completing this study by July 2019. Results will be examined at that time.

## Discussion

Online social networks hold great potential for delivering lifestyle weight loss interventions for overweight and obese adults. Delivering such interventions through online social networks overcomes many of the barriers of traditional intervention modalities and provides several distinct advantages. First, online social networks address common barriers to participation such as scheduling, transportation, weather, and childcare [2,4,5]. Second, using online social networks overcomes implementation barriers by eliminating the need and costs associated with obtaining physical space for visits, by not limiting patient pool due to daytime work schedules (eg, in-person intervention groups typically must be scheduled on weekday evenings or early mornings, limiting the number of time slots available to treat patients in a week), and by more efficiently using dietitian time. Third, online social networks are highly conducive to increased and immediate (ie, same day) feedback from peers and interventionists. Behavioral theory has long shown that the longer feedback is delayed, the less impact it has on behavior [56]. Fourth, the online social modality may be more conducive to participants building a sustainable social network to support their weight loss journey after the intervention ends. Despite this promise, a limited scientific literature has assessed the efficacy of lifestyle interventions delivered via social network. This study will be among the first to do so. Another strength of this study is the use of targeted recruitment efforts to recruit males and racial/ethnic minority participants. Males have been historically difficult to recruit in behavioral weight loss trials, and racial/ethnic minorities, especially males, are underrepresented in such studies. A review of 244 RCTs of behavioral weight loss interventions indicated that on average, 27% of study samples were male, and 1.8% of participants in US studies were racial/ethnic minority males [22]. Targeted recruitment methods such as the ones proposed in this study are needed to enhance gender and racial/ethnic diversity of study samples and generalizability of study findings.

Limitations of the methods described are as follows. First, it is possible that the time required to deliver intervention via online social networks is the same or more than that via the traditional modality. Being available daily, albeit for short periods of time, may be burdensome for interventionists in different ways than the traditional model. Second, participants in the study must be available to participate in either modality and thus may not represent people who are unable to attend frequent clinic visits, the very people who stand to benefit the most from the social network modality. If hypotheses are confirmed, the next step in this work should be to test the social network–delivered intervention in real-world settings and with patients who are unable to participate in the traditional modality or who strongly prefer an online intervention. Third, use of a social media platform that not all participants are familiar with introduces a learning curve that could be burdensome to some. Difficulties adopting a new technology could impact engagement to the extent that individuals do not prefer the interface, do not already have a habit of using it, or have difficulty understanding the interface (eg, among Twitter users, toggling to a different account might present a barrier to engagement to the extent they forget to switch to the new account). Future research should explore leveraging different commercial social media platforms in the context of behavioral intervention delivery.

Findings from this study may support an intervention delivery modality that is conducive to settings such as worksites, health plans, and clinics that serve large populations but have limited space, staffing, and resources for traditional in-person clinic-based behavioral interventions. If this trial is successful, approaches to dissemination and implementation should be explored as well as models that further reduce interventionist burden to explore how much costs could be reduced while retaining efficacy. Behavior change programming is conducive to delivery via connected technologies such as social media; thus, if proven cost-effective and more convenient relative to traditional models, connected health models could greatly improve the impact of behavioral interventions of all kinds.

## Appendix

Multimedia Appendix 1 Peer-review report from NIH for Grant 1 R01 DK103944-01A1.

## Abbreviations

BMI: body mass index
DPP: Diabetes Prevention Program
DVD: digital versatile disc
NCI: National Cancer Institute
NHLBI: National Heart, Lung, and Blood Institute
NIDDK: National Institute of Diabetes and Digestive and Kidney Diseases
SD: standard deviation
TIPI: Ten-Item Personality Inventory
