# Supplementary material for: Clinic Versus Online Social Network–Delivered Lifestyle Interventions: Protocol for the Get Social Noninferiority Randomized Controlled Trial
Source: JMIR Res Protoc. 2017 Dec 11;6(12):e243. doi: 10.2196/resprot.8068 (PMC5742659; doi:10.2196/resprot.8068)
Supplement: Multimedia Appendix 1 [file resprot_v6i12e243_app1.pdf]

SUMMARY STATEMENT
(Privileged Communication)

PROGRAM CONTACT:
Dr. ROBERT KUCZMARSKI
(301) 451-8354
kuczmarskir@niddk.nih.gov

Release Date: **06/18/2015**

Application Number: **1 R01 DK103944-01A1**

Principal Investigator

PAGOTO, SHERRY L. PHD

Applicant Organization: UNIV OF MASSACHUSETTS MED SCH WORCESTER

Review Group: **PRDP**
Psychosocial Risk and Disease Prevention Study Section

Meeting Date: **06/08/2015**
Council: **OCT 2015**
Requested Start: **12/01/2015**

RFA/PA: **PA13-302**
PCC: **NRK OPAT**
Dual IC(s): **HL**

Project Title: **Get Social: Randomized Trial of a Social Network Delivered Lifestyle Intervention**

SRG Action: **Impact Score: 15 Percentile: 5**
Next Steps: **Visit http://grants.nih.gov/grants/next_steps.htm**
Human Subjects: 30-Human subjects involved - Certified, no SRG concerns
Animal Subjects: 10-No live vertebrate animals involved for competing appl.
Gender: **1A-Both genders, scientifically acceptable**
Minority: **1A-Minorities and non-minorities, scientifically acceptable**
Children: **1A-Both Children and Adults, scientifically acceptable**
Clinical Research - not NIH-defined Phase III Trial

Project Year	Direct Costs Requested	Estimated Total Cost
1	303,630	508,580
2	363,175	608,318
3	375,760	629,398
4	293,751	492,033
5	255,557	428,058
TOTAL	1,591,873	2,666,387

ADMINISTRATIVE BUDGET NOTE: The budget shown is the requested budget and has not been adjusted to reflect any recommendations made by reviewers. If an award is planned, the costs will be calculated by Institute grants management staff based on the recommendations outlined below in the COMMITTEE BUDGET RECOMMENDATIONS section.

1R01DK103944-01A1 PAGOTO, SHERRY

RESUME AND SUMMARY OF DISCUSSION: This application seeks funds to conduct a non-inferiority randomized controlled trial testing the efficacy of a lifestyle intervention targeting weight loss delivered using an online social network approach. This resubmitted work was highly responsive to previous critiques and now offers high impact and importantly concerns the challenging public health issues of obesity and a sedentary lifestyle. Reviewers were once again very enthusiastic about this well written work noting many strengths: the reliance on the modified DPP interventions; the exceptionally novel test of social networks to promote lifestyle changes; the exceptionally significant topic; the 18 month follow up; the excellent cost analysis proposed; the examination of acceptability; the outstanding approach featuring a non-inferiority design, the careful respondent satisfaction assessment, use of accelerometers to measure physical activity, careful control for contact and content, and the strong pilot work informing this work; the exceptional and experienced Principal Investigator with outstanding expertise in social media, research team and research environment; and outstanding innovation in the focus on the impact of social networks facilitating weight loss in both males and females. Some minor weaknesses were discussed: a minority opinion opined that only moderate innovation offered.
In sum, reviewers deemed this a high impact study that is very likely to contribute to new insights about the delivery of weight loss interventions.

DESCRIPTION (provided by applicant): Lifestyle interventions have had established efficacy for over a decade but are still not widely disseminated, largely due to high cost and patient and provider burden. Online social networks are an alternative way to deliver lifestyle counseling and delivery via this modality may virtually eliminate patient visits, the main source of cost and burden in traditional modalities. Interactions in online social networks are frequent, brief, and asynchronous because users login to their online communities during downtime during work and leisure time, or when they simply feel a need for social connection. As such, social media becomes embedded into people's daily lives. By using them we have a means to embed health behavior change programming into people's daily lives. Thus far in the literature, existing online social networks have been used as component of web- or mobile app-based lifestyle interventions but not as the primary modality for intervention delivery. The purpose of the present study is to compare a lifestyle intervention delivered entirely via private groups on the online social network Twitter to a traditional in-person group-based lifestyle intervention. We performed three pilot studies of social network-delivered interventions using Twitter and Facebook and found that this approach was both feasible and acceptable. Using a randomized trial (N=328), we will test whether a lifestyle intervention delivered via an online social network (Get Social condition) will result in a mean percent weight loss at 12 months that is not appreciably worse than the gold-standard in-person group-based lifestyle intervention (Traditional condition), i.e., the social network arm will not lose on average 2 percent less than the in-person arm. Secondary non-inferiority outcomes include weight loss at 18 months, and dietary intake and physical activity at 12- and 18-months. We also hypothesize that the Get Social condition will be less expensive than the Traditional condition. To understand for whom an online social network modality is most suited, we will test predictors of weight loss in the Get Social condition including engagement, age, sociability, neuroticism, openness, and smartphone and social network use. We hypothesize that people who are younger, more sociable, engage more on the social network, higher in neuroticism/openness, and heavier overall smartphone and social network users will lose more weight in the Get Social condition. Findings from this study may support an intervention delivery modality that is more conducive to settings like worksites, health plans, and clinics that serve large populations but have limited space, staffing, and resources for traditional in person interventions. If this efficacy trial is successful, we plan an effectiveness trial in a worksite setting to build upon our previous worksite interventions.

PUBLIC HEALTH RELEVANCE: The majority of online Americans (73 percent) now use online social networks to communicate. Online social networks may be a low-cost means of delivering weight loss interventions to people who need it most. The present study will compare the efficacy of a lifestyle intervention delivered entirely via an online social network to a traditional lifestyle intervention delivered via group meetings.

CRITIQUE 1

Significance: 2
Investigator(s): 1
Innovation: 3
Approach: 2
Environment: 1

Overall Impact: This excellent resubmitted application proposes a two group non-inferiority trial comparing traditional lifestyle intervention for obesity management to lifestyle intervention delivered via several technologies with primary outcome at 12 months and following participants to 18 months. The online intervention uses three existing technology platforms My Fitness Pal for self-monitoring, Wordpress to blog about DPP content or lessons for 24 weeks, and Twitter to deliver multiple counselor tweets or messages per day to encourage behavior change and interaction among participants. The potential to use existing technology to deliver evidence based interventions is high. Discovering effective obesity treatment alternatives to face to face treatment that are cost-effective is important. Several small scale studies provide pilot data to support the application, the team has delivered lifestyle interventions previously with success, outcomes are appropriate and methods are sound. The applicant has been responsive to the prior review and revisions to the application have strengthened the approach. Modifications include equating for contact time, providing greater attention and discussion about methods used to fostering social networks, greater detail regarding preliminary data, justification for the difference that will be considered non-inferior, addition of accelerometry to objectively measure physical activity, and added measures of social influence for weight loss among others. Moderate level of innovation reduces enthusiasm somewhat, but overall this work offers high impact.

1. Significance:

Strengths

- Online social networks have received little study in obesity treatment but are very popular among consumers.
- Comparison of an online social media based intervention to an efficacious lifestyle intervention will yield important information about whether the approach is as good as the gold standard of care.

Weaknesses

- DPP lifestyle intervention has been delivered via online treatment groups and group conference calls, and other remotely delivered behavioral weight loss involving lifestyle counselors, and compared to face to face treatment. Thus, this research question is not novel.
- Cost-effectiveness of online, counselor intensive lifestyle interventions compared with face to face interventions have been done and demonstrated little to no cost savings from the payer perspective.

2. Investigator(s):

Strengths

- The Principal Investigator is experienced with mobile technology and social media interventions to promote weight loss.
- Co-investigator support is provided by a biostatistician (Ma) and a health economist (Wang) who will lead the cost-effectiveness.

Weaknesses

- None noted.

3. Innovation:

Strengths

- Using an online social network to promote behavior change is fairly novel.

Weaknesses

- This study does not study an exclusively social media delivered intervention. The basic difference between the conditions is delivery modality, face to face or online, which is not a highly novel question.
- The opportunity to study the creation of the online social network to support weight loss and its effect on weight change appears to be missed by inclusion of such heavy involvement by lifestyle counselors.

4. Approach:

Strengths

- 2 group randomized controlled trial is proposed with long term follow-up at 18 months.
- Weight, the primary outcome, is measured in person using standard protocols, accelerometry is included and 3 days of ASA 24 dietary recalls at each assessment.
- Cost will be carefully tracked and cost-effectiveness proposed.
- Contact and content are mostly controlled between groups.

Weaknesses

- In the current design, effects of social networking are not separable from frequent counselor prompting and reinforcement. For example, the online group receives multiple daily counselor messages and reinforcement of monitoring whereas the face to face group receives this weekly.
- The intervention is described as social network delivered, but appears to be multi-component, multi-platform and includes a weekly blog post related to DPP intervention lessons, use of myFitnessPal for self-monitoring and multiple daily tweets by counselors each day (13 minutes).
- Predictors of outcome and social media engagement were included, but felt superficially integrated and underdeveloped.
- Cost effectiveness analysis seems unjustified. The applicant argues for non-inferiority not superiority so it was not clear why the online intervention will cost less than the face to face since they are matched for interventionist contact time. Additionally, this has been evaluated by Harvey Berino et al in synchronous online groups where contact time was equated and the cost was not less.

- While the pilot studies show preliminary evidence for the approach, the studies were predominantly women (~90 percent) and the current study will include 50 percent men. It is unclear if this approach will appeal to men and if they will engage with the intervention with women on social media. Alternatively, this is a strength as the current study can test the approach among men.

5. Environment:

Strengths

- The research environment at University of Massachusetts Medical School is considered strong and supportive of this work.

Weaknesses

- None noted

Protections for Human Subjects:

Acceptable Risks and/or Adequate Protections

- The study is considered low risk. Appropriate protections are described for online interaction.

Data and Safety Monitoring Plan (Applicable for Clinical Trials Only):

Acceptable

- o DSMB is included as required.

Inclusion of Women, Minorities and Children:

- Sex/Gender: Distribution justified scientifically
- Race/Ethnicity: Distribution justified scientifically
- Inclusion/Exclusion of Children under 21: Including ages < 21 justified scientifically
- Minority participation is somewhat low at 20 percent and given the proximity to Boston a metro area with adequate minority representation increasing this should be considered.

Vertebrate Animals:

Not Applicable (No Vertebrate Animals)

Biohazards:

Not Applicable (No Biohazards)

Budget and Period of Support:

Recommend as Requested

CRITIQUE 2

Significance: 2
Investigator(s): 1
Innovation: 1

Approach: 2
Environment: 1

Overall Impact: This resubmitted and revised R01 application seeks to conduct the first non-inferiority RCT which would compare outcomes obtained with a behavioral lifestyle intervention delivered via a mainstream social media network vs. a traditional group-based approach. If results are indeed comparable, interventions delivered via social media could prove to be more readily accessible and convenient to participants and cost-effective to payers, thus shifting the focus of how behavioral treatments are delivered. The approach is innovative and the potential impact high. The applicants will use a non-inferiority RCT design to compare results obtained with a traditional group behavioral lifestyle intervention against a therapist-facilitated group in a mainstream social network. A total of 328 adults will be randomly assigned to the two conditions. The investigators hypothesize that at 12 months, mean percent weight loss not be significantly worse (2 percent) in the social media group. The multidisciplinary investigative team is very strong and well qualified to conduct this work. Pilot data are compelling. The research plan is well specified with careful attention to process and outcomes. Cost effectiveness analyses from the perspectives of program and participants are also included. As the applicants have adequately addressed the few minor concerns raised in the initial review, the resubmission is judged to be highly responsive to previous reviews. Given the innovation, strength of the approach and potential impact, enthusiasm is very high and unqualified.

1. Significance:

Strengths

- If therapist facilitated behavioral programs can be effectively delivered via mainstream social networks, this could significantly reduce participant burden, leverage new opportunities for social sustained support, potentially appeal to broader groups, and be more cost effective. This could shift how programs are implemented across a range of health behaviors.

Weaknesses

- None noted

2. Investigator(s):

Strengths

- The investigative team is outstanding with expertise directly relevant to the proposed work including behavioral weight loss trials, coaching via social media, epidemiology, biostatistics and health economics

Weaknesses

- None noted

3. Innovation:

Strengths

- Therapist-facilitated group behavioral treatment delivered via an online-social network has not been tested and could offer new directions for developing convenient, cost effective treatments with broad appeal

Weaknesses

- None noted

4. Approach:

Strengths

- Non-inferiority RCT comparing traditional gold standard group-based DPP program against new delivery format
- Compelling preliminary data
- Groups balanced for time
- Assessment of relevant moderators, mediators, outcomes with careful attention to process
- Accelerometry will be used to estimate physical activity
- Detailed tracking of social media use including engagement with therapists vs. peers
- 18 month follow-up evaluation
- Well specified analytic plan with strong cost effectiveness component assessing both program and participant costs

Weaknesses

- Recruitment and retention of 50 percent males is ambitious. Though a thoughtful plan is presented on how the applicants will do this, this is still some concern that men may not be as interested in participating in the traditional group behavioral arm leading to differential attrition (minor)
- Unclear whether physical activity will be tracked over 7 vs. 14 days at each measurement period (minor)

5. Environment:

Strengths

- The environment at U Mass Medical School is very strong and well suited to support the proposed work

Weaknesses

- None noted

Protections for Human Subjects:

Acceptable Risks and/or Adequate Protections

- Potential risks and adequate precautions are outlined

Data and Safety Monitoring Plan (Applicable for Clinical Trials Only):

Acceptable

- o Dr. Ma will oversee data monitoring activities. Safety monitoring will be conducted by the Principal Investigator. Although there are plans for DSMB with member(s) external to the project, more information on this aspect of the application would have been helpful.

Inclusion of Women, Minorities and Children:

- Sex/Gender: Distribution justified scientifically

- Race/Ethnicity: Distribution justified scientifically
- Inclusion/Exclusion of Children under 21: Including ages < 21 justified scientifically
- Participants will be 50 percent male, ages 18-65, with a target goal of 20 percent minorities which is reflective of local residents

Vertebrate Animals:

Not Applicable (No Vertebrate Animals)

Biohazards:

Not Applicable (No Biohazards)

Resubmission:

- This revised application retains the considerable strengths of the original submission. Additional information has been provided, a biostatistician has been added to the team, contact between groups is now balanced, procedures are outlined to strengthen peer-to-peer engagement, accelerometers will be used to track physical activity, follow-up has been extended to 18 months; these changes directly address concerns identified by previous reviewers.

Budget and Period of Support:

Recommend as Requested

Additional Comments to Applicant (Optional):

- This application was very well written and was a pleasure to read.

CRITIQUE 3

Significance: 1
Investigator(s): 2
Innovation: 1
Approach: 1
Environment: 1

Overall Impact: This resubmitted application proposes to conduct a non-inferiority 2-group RCT testing the efficacy of an online social network (Twitter) lifestyle weight loss approach compared to a traditional in-person lifestyle weight loss group. It will specifically examine whether the online approach has a mean percent weight loss at 12 months that is not appreciably worse (within 2 percent) than the traditional in-person group (gold standard). Secondary outcomes include weight loss at 18 months and dietary intake and PA at 12 and 18 months. A cost analysis will also be conducted, and predictors of weight loss in the social network condition will be examined to determine individuals most likely to be successful in the online approach. The investigators were responsive to the previous review. The environment is supportive, and the investigators have the expertise to conduct the proposed research. Although others have investigated social media as a behavior change mode, the proposed research has components that enhance its level of innovation. The primary significance of this research is that it has the potential to identify an effective intervention mode with a high potential for dissemination, thus

enhancing its potential reach and public health impact. This application has numerous strengths, with only minor weaknesses. This research has the potential to make important contributions to the field.

1. Significance:

Strengths

- Effective, cost-effective, convenient weight loss interventions are needed to reduce obesity and related health risks
- Interventions delivered social media have shown promise and have the potential to overcome key issues related to broad dissemination – cost, convenience, acceptability

Weaknesses

- None noted

2. Investigator(s):

Strengths

- Dr. Pagoto is a licensed clinical psychologist; she has expertise in behavioral tx for obesity using the DPP Lifestyle Intervention approach; she also has experience using technology-based approaches and social media for behavior change; she is an experienced NIH investigator as Principal Investigator and Co-Investigator.
- Drs. Pagoto and members of the investigative team have a history of collaboration

Weaknesses

- There is some concern that Dr. Pagoto is Principal Investigator of 5 existing grants and Co-Investigator of 6, leading to concerns that she will not have adequate time to devote to the proposed research
- There is no LOS from Dr. Wang, a consultant on the proposed research
- Dr. Wang does not appear to have experience/expertise in cost analysis of online programs; although the introduction states that he is currently conducting cost analysis for an online program (#22), his biosketch does not support this

3. Innovation:

Strengths

- Conducting a non-inferiority RCT to compare the social media approach to the gold standard
- Examining proposed predictors of success to determine for whom this approach is likely to be most effective
- Examination of the type of engagement that should be promoted in future studies to enhance successful weight loss
- Conducting a cost analysis comparing the online vs traditional arms
- Examination of maintenance effects
- Integration of behavior analytic theory and captology to inform the conceptual model underlying the proposed research

Weaknesses

- Others have examined social media as a behavior change modality

4. Approach:

Strengths

- The proposed research is a logical extension of Dr. Pagoto's research
- Pilot data supporting feasibility and acceptability of the online social network approach
- Reasonable tolerance level to compare effectiveness of two approaches (2 percent difference in weight loss)
- Secondary noninferiority outcomes – weight loss at 18 mo and diet, physical activity at 12 and 18 mo
- Cost analysis of the two arms
- Exploratory analysis to identify individuals for whom the social media approach is most likely to be effective
- Focus on theory (behavior analytic theory; captology)
- A specific plan for enrolling males in the study is provided

Weaknesses

- Unclear how engagement fits into the conceptual model presented on pg 60
- No evidence is presented that engagement (posts, replies, likes) may be linked to successful weight loss – i.e., what is the foundation for this exploratory hypothesis - there appears to be a strong emphasis on engagement and its predictors in the proposed research; therefore, it would be interesting to know the investigators' reasons for this focus (3A8)

5. Environment:

Strengths

- The environment has the resources to support the proposed research

Weaknesses

- No concerns

Protections for Human Subjects:

Acceptable Risks and/or Adequate Protections

Data and Safety Monitoring Plan (Applicable for Clinical Trials Only):

Acceptable

- DSMB planned

Inclusion of Women, Minorities and Children:

- Sex/Gender: Distribution justified scientifically
- Race/Ethnicity: Distribution justified scientifically
- Inclusion/Exclusion of Children under 21: Including ages < 21 justified scientifically

- Equal distribution of males/females 18-65 year old; minorities will be included in proportions similar to the demographics of the county where the research will be conducted (20 percent)

Vertebrate Animals:

Not Applicable (No Vertebrate Animals)

Biohazards:

Not Applicable (No Biohazards)

Resubmission:

- The investigators were highly responsive to the previous review

Budget and Period of Support:

Recommend as Requested

THE FOLLOWING SECTIONS WERE PREPARED BY THE SCIENTIFIC REVIEW OFFICER TO SUMMARIZE THE OUTCOME OF DISCUSSIONS OF THE REVIEW COMMITTEE, OR REVIEWERS' WRITTEN CRITIQUES, ON THE FOLLOWING ISSUES:

PROTECTION OF HUMAN SUBJECTS (Resume): ACCEPTABLE. There are no concerns.

INCLUSION OF WOMEN PLAN (Resume): ACCEPTABLE. Both genders will be included.

INCLUSION OF MINORITIES PLAN (Resume): ACCEPTABLE. All race and ethnic groups will be recruited.

INCLUSION OF CHILDREN PLAN (Resume): ACCEPTABLE. The sample will be comprised of children aged 18-20 and adults aged 21 to 65.

COMMITTEE BUDGET RECOMMENDATIONS: The budget was recommended as requested.

NIH has modified its policy regarding the receipt of resubmissions (amended applications). See Guide Notice NOT-OD-14-074 at http://grants.nih.gov/grants/guide/notice-files/NOT-OD-14-074.html. The impact/priority score is calculated after discussion of an application by averaging the overall scores (1-9) given by all voting reviewers on the committee and multiplying by 10. The criterion scores are submitted prior to the meeting by the individual reviewers assigned to an application, and are not discussed specifically at the review meeting or calculated into the overall impact score. Some applications also receive a percentile ranking. For details on the review process, see http://grants.nih.gov/grants/peer_review_process.htm#scoring.

MEETING ROSTER

Psychosocial Risk and Disease Prevention Study Section
Risk, Prevention and Health Behavior Integrated Review Group
CENTER FOR SCIENTIFIC REVIEW
PRDP
June 08, 2015 - June 09, 2015

CHAIRPERSON

EPSTEIN, LEONARD H, PHD
SUNY DISTINGUISHED PROFESSOR
DEPARTMENT OF PEDIATRICS
DIVISION OF BEHAVIORAL MEDICINE
STATE UNIVERSITY OF NEW YORK AT BUFFALO
BUFFALO, NY 14214

MEMBERS

ABRANTES, ANA M, PHD
ASSOCIATE PROFESSOR
DEPARTMENT OF PSYCHIATRY AND HUMAN BEHAVIOR
ALPERT MEDICAL SCHOOL
BUTLER HOSPITAL
BROWN UNIVERSITY
PROVIDENCE, RI 02906

ALLISON, KELLY C, PHD *
ASSOCIATE PROFESSOR
DEPARTMENT OF PSYCHIATRY
WEIGHT AND EATING DISORDERS PROGRAM
UNIVERSITY OF PENNSYLVANIA
PHILADELPHIA, PA 19104

BAEZCONDE-GARBANATI, LOURDES A, MPH, PHD
ASSOCIATE PROFESSOR
DEPARTMENT OF PREVENTIVE MEDICINE
AND SOCIOLOGY, KECK SCHOOL OF MEDICINE
NORRIS COMPREHENSIVE CANCER CENTER
UNIVERSITY OF SOUTHERN CALIFORNIA
LOS ANGELES, CA 90033

BARTLETT, SUSAN J, PHD
ASSOCIATE PROFESSOR
DIVISION OF CLINICAL EPIDEMIOLOGY
ROYAL VICTORIA HOSPITAL
MCGILL UNIVERSITY
MONTREAL, QC H3A 1A1
CANADA

BASKIN, MONICA L, PHD
PROFESSOR
DIVISION OF PREVENTIVE MEDICINE
DEPARTMENT OF HEALTH BEHAVIOR
UNIVERSITY OF ALABAMA AT BIRMINGHAM
BIRMINGHAM, AL 35294

BOHNERT, AMY , PHD *
ASSOCIATE PROFESSOR
DEPARTMENT OF PSYCHOLOGY
LOYOLA UNIVERSITY OF CHICAGO
CHICAGO, IL 60626

BOUTELLE, KERRI N, PHD
PROFESSOR
DEPARTMENT OF PEDIATRICS
AND PSYCHIATRY
UNIVERSITY OF CALIFORNIA, SAN DIEGO
LA JOLLA, CA 92037

BUTRYN, MEGHAN , PHD *
ASSOCIATE RESEARCH PROFESSOR
DEPARTMENT OF PSYCHOLOGY
DREXEL UNIVERSITY
PHILADELPHIA, PA 19104

COUPS, ELLIOT J, PHD
ASSOCIATE PROFESSOR
DEPARTMENT OF MEDICINE
ROBERT WOOD JOHNSON MEDICAL SCHOOL
RUTGERS, THE STATE UNIVERSITY OF NEW JERSEY
NEW BRUNSWICK, NJ 08901

DONNELLY, JOSEPH E, EDD, BS, MS *
PROFESSOR
CENTER FOR PHYSICAL ACTIVITY
AND WEIGHT MANAGEMENT
UNIVERSITY OF KANSAS MEDICAL CENTER
LAWRENCE, KS 66045

FERRY, ROBERT , MD, BS *
PROFESSOR
DEPARTMENT OF PEDIATRICS
DIVISION OF PEDIATRIC ENDOCRINOLOGY
UNIVERSITY OF TENNESSEE HEALTH SCIENCES
CENTER
MEMPHIS, TN 38103

FISCELLA, KEVIN , MPH, MD
PROFESSOR
DEPARTMENT OF FAMILY MEDICINE
SCHOOL OF MEDICINE
UNIVERSITY OF ROCHESTER
ROCHESTER, NY 14620

FITZPATRICK, STEPHANIE L, PHD *
ASSISTANT PROFESSOR
DEPARTMENT OF PREVENTIVE MEDICINE
RUSH UNIVERSITY MEDICAL CENTER
CHICAGO, IL 60612

FRANCIS, LORI A, PHD *
ASSOCIATE PROFESSOR
DEPARTMENT OF BIOBEHAVIORAL HEALTH
PENNSYLVANIA STATE UNIVERSITY
UNIVERSITY PARK, PA 16802

FRASER, CLAIRE M, PHD *
PROFESSOR AND DIRECTOR
INSTITUTE OF GENOME SCIENCES
UNIVERSITY OF MARYLAND SCHOOL OF MEDICINE
BALTIMORE, MD 21201

HODGE, FELICIA S, DRPH *
PROFESSOR
SCHOOL OF NURSING AND PUBLIC HEALTH
UNIVERISITY OF CALIFORNIA, LOS ANGELES
LOS ANGELES, CA 90095

LEAHEY, TRICIA M, PHD
ASSOCIATE PROFESSOR
DEPARTMENT OF ALLIED HEALTH SCIENCES
UNIVERSITY OF CONNECTICUT
STORRS, CT 062991101

LEVINE, MICHELE D, PHD
ASSOCIATE PROFESSOR
DEPARTMENT OF PSYCHIATRY
UNIVERSITY OF PITTSBURGH
PITTSBURGH, PA 15213

MORLAND, KIMBERLY B, MPH, PHD
ASSOCIATE PROFESSOR
DEPARTMENT OF PREVENTIVE MEDICINE
ICAHN SCHOOL OF MEDICINE
AT MOUNT SINAIL
NEW YORK, NY 10029

NEELON, SARA E, PHD *
ASSOCIATE PROFESSOR
DEPARTMENT OF HEALTH,
BEHAVIOR AND SOCIETY
JOHNS HOPKINS SCHOOL OF PUBLIC HEALTH
BALTIMORE , MD 21205

PARRA-MEDINA, DEBORAH , PHD *
PROFESSOR
DEPARTMENT OF EPIDEMIOLOGY AND BIOSTATISTICS
SCHOOL OF MEDICINE
UT HEALTH SCIENCES CENTER AT SAN ANTONIO
SAN ANTONIO, TX 78229

PAUL, IAN M, MD
PROFESSOR
DEPARTMENT OF PEDIATRICS
COLLEGE OF MEDICINE
PENNSYLVANIA STATE UNIVERSITY
HERSHEY, PA 17033

QUARELLS, RAKALE C, PHD
ASSOCIATE PROFESSOR
CARDIOVASCULAR RESEARCH INSTITUTE
MOREHOUSE SCHOOL OF MEDICINE
ATLANTA, GA 30310

SPRING, BONNIE , PHD
PROFESSOR
DEPARTMENT OF PREVENTIVE MEDICINE
NORTHWESTERN UNIVERSITY
CHICAGO, IL 60611

SULLIVAN, DEBRA K, PHD *
PROFESSOR AND CHAIR
DEPARTMENT OF DIETETICS AND NUTRTION
UNIVERSITY OF KANSAS MEDICAL CENTER
KANSAS CITY, KS 66160

TATE, DEBORAH F, PHD
ASSOCIATE PROFESSOR
DEPARTMENTS OF HEALTH
BEHAVIOR AND NUTRITION
UNIVERSITY OF NORTH CAROLINA
CHAPEL HILL, NC 27599

THOMPSON, DEBORAH I, PHD
ASSOCIATE PROFESSOR OF PEDIATRICS
USDA/ARS SCIENTIST/NUTRITIONIST-CHILD OBESITY
CHILDREN'S NUTRITION RESEARCH CENTER
BAYLOR COLLEGE OF MEDICINE
HOUSTON, TX 77030

VERSALOVIC, JAMES , PHD *
PROFESSOR AND VICE CHAIR
DEPARTMENT OF PATHOLOGY
TEXAS CHILDREN'S HOSPITAL
FEIGIN CENTER
HOUSTON, TX 77030

WILFLEY, DENISE E, PHD
PROFESSOR
DEPARTMENT OF PSYCHOLOGY
WASHINGTON UNVIVERSITY AT ST LOUIS
ST LOUIS, MO 63110

WOLIN, KATHLEEN Y, SCD, BA *
ASSOCIATE PROFESSOR
COEUS HEALTH
CHICAGO, IL 60654

MAIL REVIEWER(S)

BENNETT, GARY G, PHD
PROFESSOR OF PSYCHOLOGY
DEPARTMENT OF GLOBAL
HEALTH AND MEDICINE
DUKE UNIVERSITY
DURHAM, NC 27705

SMITH, MICHAEL T, PHD
PROFESSOR
DEPARTMENT OF PSYCHIATRY, AND BEHAVORAL SLEEP
MEDICINE PROGRAM, CENTER FOR BEHAVIOR
AND HEALTH SCHOOL OF MEDICINE
BAYVIEW MEDICAL CENTER JOHNS HOPKINS
UNIVERSITY
BALTIMORE, MD 21224

SCIENTIFIC REVIEW OFFICER

FITZSIMMONS, STACEY , MPH, PHD
SCIENTIFIC REVIEW OFFICER
CENTER FOR SCIENTIFIC REVIEW
NATIONAL INSTITUTES OF HEALTH
BETHESDA, MD 20892

EXTRAMURAL SUPPORT ASSISTANT

WATTS, MELISSA D
EXTRAMURAL SUPPORT ASSISTANT
CENTER FOR SCIENTIFIC REVIEW
NATIONAL INSTITUTE FOR HEALTH
BETHESDA, MD 20892

* Temporary Member. For grant applications, temporary members may participate in the entire meeting or may review only selected applications as needed.

Consultants are required to absent themselves from the room during the review of any application if their presence would constitute or appear to constitute a conflict of interest.
